# The association between patient safety culture and adverse events – a scoping review

**DOI:** 10.1186/s12913-023-09332-8

**Published:** 2023-03-29

**Authors:** Magnhild Vikan, Arvid Steinar Haugen, Ann Kristin Bjørnnes, Berit Taraldsen Valeberg, Ellen Catharina Tveter Deilkås, Stein Ove Danielsen

**Affiliations:** 1 Department of Nursing and Health Promotion, Faculty of Health Sciences, Oslo Metropolitan University, Oslo, Norway; 2 Department of Anaesthesia and Intensive Care, Haukeland University Hospital, Bergen, Norway; 3 University of South-Eastern Norway, Drammen, Norway; 4 Akershus University Hospital, Lørenskog, Norway

**Keywords:** Patient safety culture, Patient safety climate, Organisational safety culture, Adverse events, Patient outcomes, Medical errors

## Abstract

**Background:**

Adverse events (AEs) affect 10% of in-hospital patients, causing increased costs, injuries, disability and mortality. Patient safety culture (PSC) is an indicator of quality in healthcare services and is thus perceived as a proxy for the quality of care. Previous studies show variation in the association between PSC scores and AE rates. The main objective of this scoping review is to summarise the evidence on the association between PSC scores and AE rates in healthcare services. In addition, map the characteristics and the applied research methodology in the included studies, and study the strengths and limitations of the evidence.

**Methods:**

We applied a scoping review methodology to answer the broad research questions of this study, following the PRISMA-ScR checklist. A systematic search in seven databases was conducted in January 2022. The records were screened independently against eligibility criteria using Rayyan software, and the extracted data were collated in a charting form. Descriptive representations and tables display the systematic mapping of the literature.

**Results:**

We included 34 out of 1,743 screened articles. The mapping demonstrated a statistical association in 76% of the studies, where increased PSC scores were associated with reduced AE rates. Most of the studies had a multicentre design and were conducted in-hospital in high-income countries. The methodological approaches to measuring the association varied, including missing reports on the tools` validation and participants, different medical specialties, and work unit level of measurements. In addition, the review identified a lack of eligible studies for meta-analysis and synthesis and demonstrated a need for an in-depth understanding of the association, including context complexity.

**Conclusions:**

We found that the vast majority of studies report reduced AE rates when PSC scores increase. This review demonstrates a lack of studies from primary care and low- and- middle-income countries. There is a discrepancy in utilised concepts and methodology, hence there is a need for a broader understanding of the concepts and the contextual factors, and more uniform methodology. Longitudinal prospective studies with higher quality can enhance efforts to improve patient safety.

**Supplementary Information:**

The online version contains supplementary material available at 10.1186/s12913-023-09332-8.

## Background

‘Adverse events’ (AE) harm and impact the lives of the involved patients, families and healthcare professionals, and AEs are one of the leading causes of disability and death in the world [1]. Patient AEs can be defined as unintended actions or omission that lead to or can lead to harm or injuries related to healthcare and not to the underlying disease [2]. AEs occur in at least 10% of in-hospital patients [1, 3, 4], and half of the AEs are estimated to be preventable [4–7]. AEs in low- and middle-income countries are estimated to affect 25% of hospitalised patients, constituting 134 million AEs and 2.6 million deaths annually [1, 5, 8]. The World Health Organization estimates the global costs of AEs to be US$ 1–2 trillion a year [5]. In Organization for Economic Co-operation and Development countries, 15% of all hospital costs are due to patient harm from AEs [1, 5].

Estimates of the occurrence of AEs as patient outcomes are often based on measurements from medical record reviews [4]. The ‘Harvard Medical Practice Study’ (HMPS) and the’Global Trigger Tool’ (GTT) are the most frequently used structured chart review methods for measuring AE rates in electronic medical records. The occurrence of in-hospital AE rates varies across studies, ranging from 2.9–21.9%, indicating methodological and contextual variation in retrospective chart reviews, as well as varying levels of patient safety [4]. A comprehensive approach to monitoringand learn from AEs is important to improve patient safety in healthcare systems [1, 4, 8, 9].

‘Safety culture’ is a multidisciplinary concept, however the interest of the concept safety culture in healthcare organisations, as ‘Patient safety culture’ (PSC) increased after the Chernobyl disaster in 1986, and PSC is perceived as a proxy outcome for quality of care [10–12]. PSC can be described as the overall attitudes and patterns of behaviours related to the patient safety work at multiple levels in an organisation. This includes individuals and groups` shared values, beliefs and norms influencing their actions both in preventing AEs in care delivery, and when an AE occurs [13–15]. In addition, the organisation`s efforts to protect patients from AEs through communication openness, organisational learning and error management culture are important dimensions influencing the PSC [12]. Another important dimension of PSC is the ‘Patient safety climate’. ‘Patient safety climate’ is also used interchangeable to PSC, and ‘Patient safety climate` can be considered as the more visible perceptions and deliberate behaviours. This can be measured, and indicates the priority given to safety at different healthcare levels, hence the PSC can also represent the invisible and intangible underlying dimensions. In this review, ‘Safety culture’ and ‘Safety climate’ in healthcare services will be covered by the concept PSC.

Measuring PSC has the last two decades become a strategy for understanding the processes of care and improving the overall quality of healthcare [16]. A questionnaire is often the tool used for such measurements, and a previous review of PSC measurement totally identified 127 tools. The review identified 11 main dimensions of PSC across the studied tools, however no single tool captured all the dimensions [17]. The most reported dimensions from such measurements are ‘Leadership’, ‘Perception of safety’, ‘Teamwork and collaboration’, ‘Safety systems’, ‘Prioritisation of safety’ and ‘Resources and constraints’ [17]. Healthcare should be aware of choosing an appropriate and validated tool [16]. The Safety Attitudes Questionnaire (SAQ) and the Agency for Healthcare Research and Quality Hospital Survey on Patient Safety Culture (HSOPS) are validated, and the tools used most frequently to measure PSC [16–18]. A recent review investigating PSC instruments for measurements in hospital settings, suggests that valid measurements of PSC can identify variability in healthcare professionals shared perceptions and guide the management to focus on the challenging PSC dimensions in their organisation [16].

Groves [19] reviewed the evidence on the association between safety culture and patient safety outcomes in acute medical care Despite a variety of concepts of safety culture and outcomes, work level of measurement and instruments utilised, ten out of fourteen studies were included in a meta-analysis. No significant association was reported in this review from 2013 [19]. DiCurrio [20] investigated the association between PSC scores and nurse-sensitive outcomes in hospitals. This review reported that a limited number of the included studies found a statistically significant association.However, this review limited the measurement to only selected nurse-sensitive outcomes in hospitals and excluded reports from healthcare professionals` perceptions [20]. Braithwaite [21] reviewed the association between the wide-range concepts of ‘Organisational- and workplace cultures’, and a broad range of included patient outcomes. This review from 2017 demonstrated a statistically significant inverse association in the included evidence and pointed to the need for higher quality studies to verify the findings [21].

We need to gain a better understanding of the association between PSC scores and AE rates [19–22], and an updated review is needed due to the rapid development of evidence in this research field. The main objective of this scoping review is to summarise the evidence on the association between PSC scores and AE rates in healthcare services. Moreover, in addition, map the characteristics and applied research methodology in the included studies and study the strengths and limitations of the evidence.

## Methods

### Scoping review

We conducted a scoping review to answer the main objectives. Scoping reviews often map and describe the available evidence and key concepts on a topic [23, 24]. The research questions in scoping reviews address broader topics than research questions in traditional systematic reviews. A scoping review may be the appropriate approach when a research question is complex, and a comprehensive review of the topic has yet to be undertaken [23, 24]. A scoping review is an appropriate methodological approach due to the broad objective to investigate the evidence on the association, and to scope a body of the literature [24]. We used the PRISMA-ScR (Preferred Reporting Items for Systematic reviews and Meta-Analyses extension for Scoping Reviews) checklist and explanation for complete and transparent reporting of the scoping review [25]. We developed a study protocol prior to the literature searches. The protocol was not registered, however there were no deviations from the protocol. The filled PRISMA-ScR checklist and the study protocol are available in supplemental materials.

### Search strategy

A comprehensive search strategy covering relevant databases was developed in collaboration with an experienced research librarian. The search terms and the three-step search strategy in Medline (Ovid) is outlined in Table 1, and the inclusion and exclusion criteria in Table 2. When conducting step 2 and 3, saved hits from previous steps were excluded. Despite using “patient safety culture” and “adverse patient events” in this study, we searched for a wide range of concepts to capture relevant evidence. We had no exclusion criteria on publication year due to the purpose of the broad objectives. We conducted the search in the databases Embase, PsycINFO, Cinahl, Cochrane Library and Epistemonikos in accordance with the different database thesauruses. The interprofessional databases Web of Science and Business Source Elite were also searched to identify relevant studies conducted in organisational and management research. In these databases, we subsequently narrowed the search by not using proximity operators. For a search across a greater variety of resources to possibly find pertinent evidence to supplement to supplement the primary search, we replicated the search in Google scholar, however we found no new hits after the first sixty results.

**Table 1:** The search strategy in Medline (Ovid)

Main search	January 2021	*((patient safety) adj10 culture).tw,kw,kf AND medical errors* (MeSH) OR adverse event* OR adverse fail* OR adverse outcome* OR clinical complications* limitation language
Enhanced search	March 2021 and January 2022	*((Patient adj10 safety adj10 culture) OR safety climate OR organizational culture OR safety management).tw,kw,kf. AND medical errors* (MeSH) OR adverse event* OR adverse outcome* OR clinical complications* OR postoperative complications OR mortality OR morbidity.tw,kw,kf

**Table 2:** Inclusion and exclusion criteria

Inclusion Criteria	Language: English or Scandinavian
	Measurement of PSC and AE and the association between these variables
	Peer-reviewed articles of single studies empirically measuring the association
	No limitations year
Exclusion Criteria	Conference abstracts and disseminations
	Not peer-reviewed

### Selection of sources

The records retrieved were first screened by MV and SOD independently based on titles and abstracts. To ensure the process and conduct a blind screening, we used the Rayyan software as a screening tool [26]. We tested screening agreement after the first, second and third hundred records to ensure and align a common understanding of the studies that illuminated the scope of the review. The inter-rater agreement was 95%. In the event of conflict on inclusion, consensus was achieved by discourse. After screening, we included records for full text reading. This part of the screening was also blinded by Rayyan [26]. We conducted backward citation checking in the reference lists of included studies and in relevant reviews [27, 28].

### Data charting, critical appraisal and synthesis of results

The data charting was performed using a pre-formatted Excel worksheet (Microsoft, Redmond, WA, USA, version: 2018). We charted first author, year, origin, purpose, single or multicentre study, medical specialty, intervention, quality improvement project or not, tools used to measure PSC and AEs, participants, response rate, AEs measured, the work unit level of measurement, analysis methods, time frame, confounding variables, key findings and reported knowledge gaps. Moreover, the measurement of PSC, AEs and statistical associations were specified in detail in the respective worksheets.

We explored the methodological quality of the included studies due to the heterogeneity of the individual sources of evidence, and conducted a critical appraisal of the included studies utilising the appropriate checklist ‘Quality Assessment Tool for Observational Cohort and Cross-Sectional Studies’ [29]. The tool consists of 14 questions designed to evaluate the internal validity of studies and consider the potential risk of bias. MV and SOD conducted a consensus process to assess the risk of bias in the included studies, and translated the overall risk as ‘Good’, ‘Fair’ or ‘Poor’ [29]. The complete data charting form and critical appraisal is available in supplemental materials.

We summarised, made table schema maps and descriptive representations of the charted data relating to the research questions and study objectives.

## Results

### Selection and characteristics of sources of evidence

**Fig. 1:**
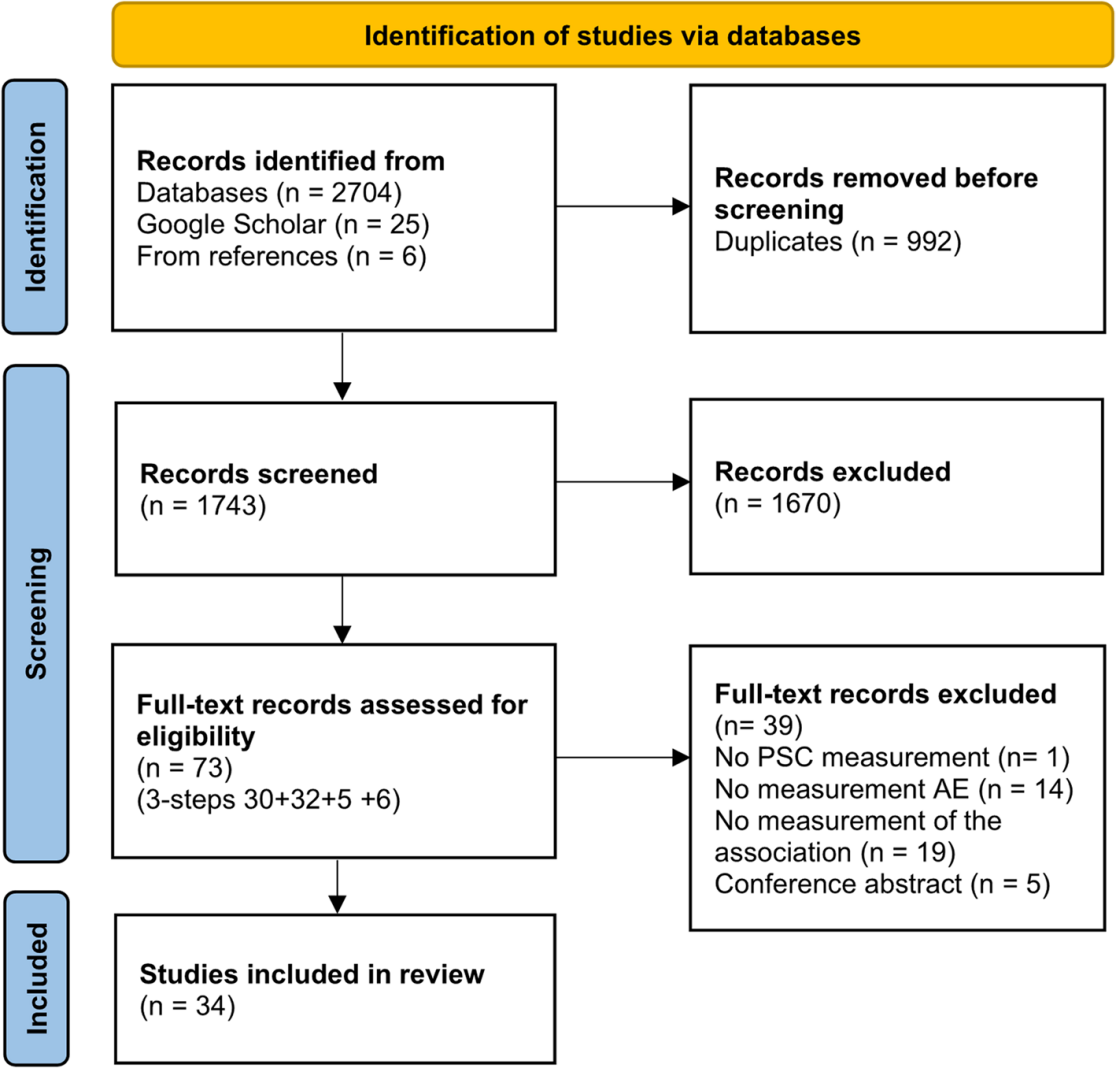
PRISMA flow chart on inclusion of studies

The selection of sources of evidence is presented by PRISMA flow chart [30] in Fig. 1. Fifteen per cent of the included studies were published within the last two years, 74% of the studies within the last decade and 12% of the studies were published in 2006–2009. Sixty-two per cent of the studies originated from the US and Canada, 21% from Europe, 15% from Asia and 3% from Australia. Most of the studies, 97%, were conducted as multicentre studies, and 91% of the studies were conducted in-hospital, otherwise in out-patient or primary care [31–33]. Surgery was the most frequently assessed medical context in hospitals, assessed in 32% of the studies. See Table 3 for more details about characteristics.

**Table 3:** The measurements of patient safety culture and adverse patient events, and their associations

First author -Year -Origin -Concepts PSC/ AE	PSC -Tool -Dimensions/items -Context -Participants PSC -*n* = -Response rate %	AE -Method -Number patients/records -Numbers and types of AEs assessed	Analysis -Methods -Study level -Number of units	Reported association Statistical values	Critical appraisal Quality Assessment Tool for Observational Cohort and Cross-Sectional Studies [29]
Ausserhofer (2013) Swiss [34] Patient Safety Climate/ Patient Outcomes	SOS 1/9 (complete) –translated Surgery/medicine/ mixed Nurses *n* = 1,630 Response rate: 72%	Retrospective nurse-estimate Incidence of AEs last year – 7-point scale 1,630 nurses 7 AEs: Nurse-reported medication errors, pressure ulcers, patient falls, urinary tract infection, bloodstream infection, pneumonia (and patient satisfaction)	Bivariate and multivariate logistic regressions Unit and hospital level 132 units in 35 hospitals	**No statistically significant associations** between PSC scores and 6 selected AEs: pressure ulcers, urinary tract infection, bloodstream infection, medication errors, pneumonia, patient falls (nor any association between PSC and patient satisfaction) Increased scores of Rationing of nursing care were consistently associated with increased rates of ^b^bloodstream infection, ^b^medication administration error, ^c^pneumonia (and ^d^patient satisfaction) in multivariate analysis (Reduced nurse ratio increases AE rates) ^a^*p* = 0.004 ^b^*p* = 0.026 ^c^*p* = 0.027 ^d^*p* = 0.005	**Fair**
Bacon (2021) US [35] Organisational Safety Climate/ Mortality and Failure-to-rescue	Safety Climate Tool – revised (originally Zohar`s measure of safety climate) 0/33 Surgery Nurses, physicians, others *n* = 261 Response rate: NR	Chart review AHRQ Quality Indicators 10,823 patients 52,898 records 2 AEs: Failure-to-rescue (deep vein thrombosis/ pulmonary embolus, pneumonia, sepsis, cardiac arrest, hemorrhage) and in-hospital mortality	Multilevel models Bonferroni correlation Hospital level 2 hospitals	**No statistically significant associations** between PSC scores and rates of in-hospital mortality or failure-to-rescue Reported in *p*-values	**Fair**
Birkmeyer (2013) US [36] Safety Culture/ Complications	HSOPS + SAQ-OR + questions disruption 4/30 Surgery Surgeons, nurses/ operating room technicians, operating room administrators *n* = 184 Response rate: surgeons 95% nurses/ operating room technicians 82%, operating room administrators 68%	Chart review Standardised instrument 24,117 patients 10 types of AEs: Abdominal abscess, bowel obstruction, leak, bleeding, respiratory failure, renal failure, wound infection/ dehiscence, venous thromboembolism, myocardial infarction or cardiac arrest, death. The AE rate is the overall rate of the 10 AEs	Bivariate regression model Spearman`s Correlation (*p*) Hospital level 22 hospitals	Increased overall PSC scores from ^a^surgeons and ^b^nurses were associated with reduced AE rates. Increased scores of ^c^Hospital safety culture by nurses and increased scores of ^d^Operating room safety by surgeons were associated with reduced AE rates ^*a*^*p* < *0.001 bp* < *0.011* ^c^*p* < *0.002* ^d^*p* < *0.045*	**Fair**
Bosch (2011) Netherlands [32] Organisational Culture and Teamwork Climate/ Pressure Ulcers	Team Climate Inventory (TCI)- short + Competing Values Framework (CVF) (both translated) 0/14 + 5/20 Ward units in hospitals and nursing homes Physicians, nurses, nursing assistants *n* = 460 Response rate 41% for the hospital wards, and 39% for the nursing home wards	Prospective nurse-reporting and scoring 1,274 patients 1 AE: Nosocomial pressure ulcers	Bivariate and multilevel logistic regression (General Linear regression) Unit level 104 units	**No statistically significant association** between PSC scores, Team climate or Preventive quality management at ward level, and the prevalence of nosocomial pressure ulcers (^a^Increased scores of Institutional quality management were significantly correlated with increased scores of Preventive quality management at ward level.) ^a^*p* < 0.001	**Fair**
Brown (2013) US [37] Safety Culture/ Adverse Patient Outcomes	HSOPS 12/42 (complete) + Global rating composite (4 items) NR Nurses NR Response rate: NR	Register National Database for Nursing Quality Improvement. The Collaborative Alliance for Nursing Outcomes (CALNOC). CALNOC indicator definitions 3 AEs: Health Acquired Pressure Ulcers, reported falls and falls with injury	Linear regression, Pearson product-moment correlations (r) Unit level 9 hospitals 37 units	^a^Increased scores of Teamwork within units were associated with reduced reported falls and ^b^increased scores of Management support were associated with increased rates of reported falls ^c^Increased Global rating composite was associated with reduced rates of Health-Acquired Pressure Ulcers (HAPU) ^d^Skill mix, Staff turnover and Workload intensity are strongly corelated with PSC scores ^a^*p* < 0.05 ^b^*p* < 0.03 ^c^*p* < 0.05 ^d^*p* < *0.01–0.05*	**Poor**
Brubakk (2019) Norway [38] Organisational Culture/ Mortality	SAQ (and organisational factors survey) 1/2 (+ 19/ 57) Emergency/ acute care Nurses, physicians, managers *n* = 8,800 Response rate: 72% 2010 77% in 2011 75% in 2012	Register The Norwegian Institute of Public Health 46,026 admitted patients 1 AE: Risk-adjusted 7-day mortality	Multivariable regression Unit and group level 20 hospitals 56 units	^a^Reduced scores of Patient safety climate and ^b^Leadership were associated with increased 7-day mortality ^c^Increased scores of Workload perceived by nurses were associated with increased 7-day mortality rates. ^d^Increased Middle manager`s engagement levels were associated with reduced 7-day mortality rates ^a^*p* < *0.003* ^b^*p* < *0.045* ^*c*^*p* < *0.028* ^*d*^*p* < *0.037*	**Good**
Camargo (2012) US/ 20 States [39] Safety Climate/ Adverse Events and Medical Error	Survey – constructed 9/50 Emergency/ acute care Nurses, physicians *n *= 3,562 Response rate: 66%	Chart review Standardised form: 18 questions 9,821 charts 3 AE-categories: Medical error, adverse event (preventable and non-preventable) and near miss (intercepted and non-intercepted) Scaled: Significant, serious, life threatening or fatal	Multivariable regression models Incident Rate Ratio (IRR) Unit level 62 units	**No statistically significant association** between PSC scores and preventable AE rates, nor was there an association between PSC scores and serious violations of treatment guidelines ^a^Increased PSC scores were significantly associated with increased “Intercepted near misses” ^a^IRR 1.79 (1.06–3.03)	**Fair**
Davenport (2007) US [40] Organisational Safety Climate/ Outcomes	SAQ 6/30 (complete) Surgery Nurses, physicians, others *n* = 6,083 Response rate: 52%	Chart review NSQIP protocol 57,880 patients 2 AEs: Risk-adjusted surgical morbidity (patient having 1 or more out of 21 complications) and 30-day mortality	Multivariate logistic regression Spearman`s* p* correlation Hospital level 52 hospitals	**No statistically significant association** between PSC scores and rates of 30-day mortality or 30-day morbidity (21 postoperative complications) ^a^Increased scores of Communication/ collaboration with doctors were correlated with reduced rates of risk-adjusted morbidity Scores of Burnout was not correlated with AE rates ^a^*p* < 0.01	**Good**
Fan (2016) US/ Minnesota [41] Safety Culture/ Surgical Outcomes	HSOPS 12/42 (complete) Surgery NR *n* = 1,926 Response rate: 43%	Prospective reporting as defined by the National Healthcare Safety Network NR 1 AE: Postoperative colon surgery SSI/ number of operations performed	Bivariate and multivariate linear regression Pearson product-moment correlations (r) Unit level 7 units/hospitals	Increased scores of following PSC dimensions were associated with reduced rates of Colon SSI: ^a^Teamwork across units ^b^Teamwork within units ^c^Organisational learning ^d^Feedback and communication about error ^e^Communication openness* ^f^Overall perception of safety ^g^Management support for patient safety ^h^Supervisor/manger expectations and actions promoting safety ^i^Non-punitive response to error* ^j^Frequency of events reported ^k^Handoffs and transitions when adjusting for ASA and surgical volume ^a^ *r *= -0.96, [-0.76, -0.99] ^b^ *r* = -0.88, [-0.38, -0.98] ^c^ *r* = -0.95, [-0.71, -0.99] ^d^ *r* = -0.92, [-0.56, -0.99] ^e^ *r* = -0.85, [-0.26, -0.98] ^f^ *r* = -0.90; [-0.45, -0.99] ^g^ *r* = -0.90, [-0.44, -0.98] ^h^ *r* = -0.85, [-0.25, -0.98] ^i^ *r* = -0.78, [-0.07, -0.97] *non-significant when adjusting for ASA and surgical volume ^j^ *r* = -0.76, [-0.01, -0.96] ^*k*^*p* < *0.05*	**Fair**
Garrouste-Orgeas (2015) France [42] Safety Culture/ Medical Error	SAQ-ICU 6/63 (complete) ICU Nurses, physicians, others *n* = 1,534 Response rate: 77.2%	Prospective reporting/ observation 8 h /5 days /2 weeks combined to chart review 4 AEs: Error administration anticoagulant medication, error prescribing anticoagulant medication, error administration insulin, accidental removal of a central venous catheter, accidental extubating	Multivariate hierarchical model Unit level 31 units	**Limited statistically significant association** between PSC scores and rates of medical errors/ patient days^a^ Increased scores of ^b^Depression symptoms, ^c^ICU organisation (40% off work previous day), ^d^Staff-specific safety training programme and ^e^Patient level/ workload were associated with increased AE rates Increased scores of Burnout were not statistically significantly correlated with increased AE rates ^*a*^*p* = *0.04–0.87* ^*b*^*p* = *0.01* ^*c*^*p* = *0.01* ^*d*^*p* = *0.001–0.02* ^*e*^*p* = < *0.0001- 0.03*	**Good**
Han (2020) South Korea [43] Patient Safety Culture/ Adverse Events	HSOPS 12/42 (complete) NR NR *n* = 212 Response rate: 86%	Retrospective nurse-estimate Incidence of AEs last year – 7-point scale 212 nurses 5 AEs: Falls, medication errors, pressure ulcers, health-associated infections (surgical site, urinary tract, central-line associated bloodstream infections and ventilator-associated pneumonia) and physical restrain ≥ 8 h (Combined into a binominal variable: “never/happened”)	Bivariate regression and multiple logistic regression. Odds Ratio (OR) Hospital level 2 hospitals	Increased scores of the following PSC dimensions were associated with reduced AE rates: ^a^Supervisor/ manger expectations and actions promoting safety and 4 AEs ^b^Communication openness and 4 AEs ^c^Management support for patient safety and 3 AEs ^d^Teamwork across units and 3 AEs ^e^Teamwork within units and 2 AEs ^f^Feedback and communication about error and 2 AEs ^g^Nonpunitive response to error and 1 AE ^h^Handoffs and transitions and 1 AE ^i^Organisational learning – continuous improvement and 1 AE Increased scores for Patient safety competencies were associated with reduced AE rates Odds Ratio (OR): ^a^ OR 0.33–0.39 ^b^ OR 0.25–0.51 ^c^ OR 0.22–0.55 ^d^ OR 0.29–0.47 ^e^ OR 0.23–0.51 ^f^ OR 0.43–0.52 ^g^ OR 2.08 ^h^ OR 2.02 ^i^ OR 0.053 ^j^ OR 0.024–0.049	**Fair**
Haynes (2011) US [44] Safety Climate/ Postoperative Morbidity and Mortality	SAQ-OR NR/6 Surgery Nurses, physicians, others *n* = 281 pre intervention *n *= 257 post intervention Response rate: 97.7%	Chart reviews and communication with clinical teams Charts were reviewed at discharge or in 30 days 19 AEs: Acute renal failure, bleeding requiring ≥ 4 units of red cell transfusion within 72 h after surgery, cardiac arrest requiring cardiopulmonary resuscitation, coma for ≥ 24 h, deep venous thrombosis, myocardial infarction, unplanned intubation, ventilator use for ≥ 48 h, pneumonia, pulmonary embolism, stroke, major wound disruption, surgical site infection, sepsis, septic shock, systemic inflammatory response syndrome, unplanned return to the OR, vascular graft failure and death	Correlation analysis Spearman`s correlation (*p*) Hospital level 8 hospitals	Increased PSC scores were associated with reduction in postoperative complication rate^a^ including 18 AE rates and mortality (The measurement was related to an intervention.) ^a^ *r* = 0.7143 *p* < 0.0381	**Fair**
Hofmann (2006) US [45] Safety Climate/ Medication Error and Patient Outcomes	Zohar`s measure of safety climate – revised and Error Orientation Scale 3/9 + 3/13 Surgery/ medicine Nurses *n* = 1,127 Response rate: NR	Chart review Coordinators collected the AE frequency over 3 months 2 AEs: Medication Errors and Urinary Tract Infections	Bivariate regression and multiple logistic regression Unit level 42 hospitals 81 units	A) Increasing overall PSC scores significantly predict reduced rates of ^a^medication errors and ^b^urinary tract infections B) Regression with^ab^show that^a^ was significantly moderated by Patient complexity A)^a^ -1.51 *p* < 0.05 ^b^ -1,27 *p* < 0.05 B) ^ab^ -7.85 *p* < 0.05	**Fair**
Huang (2010) US [46] Safety Culture/ Outcomes	SAQ-ICU 6/60 (complete) ICU Nurses, physicians, others *n* = 2,103 Response rate: 47.9%	Register PICCM clinical national database 65,978 patients 2 AEs: Hospital mortality and LOS	Linear regression model and multivariate logistic regression Unit level 30 units	^a^Increased scores of Perceptions of management were associated with reduced mortality rates ^b^Increased scores of Safety climate were associated with reduced LOS ^a^*p* = 0.005 ^b^*p* = 0.003	**Fair**
Hwang (2011) Korea [47] Safety Climate/ Medical Errors	SAQ – translated 17 items from 4 dimensions + 4 items added due to Korean context 2/21 NR Nurses *n* = 1,923 Response rate: 89.7%	Nurse-estimate experienced errors in retrospective questionnaire n = 277 nurses AEs last year: Yes/ No Frequency of AEs last year	Multiple logistic regression Hospital level 33 hospitals	Nurses with better scores of ^a^workgroup and ^b^organisation-level Safety climate were associated with reduced error rates Odds Ratio: ^a^(OR = 0.73) *p* < *0.001* ^b^(OR = 0.69) *p* < *0.001*	**Poor**
Kakemam (2021) Iran [48] Patient Safety Culture/ Adverse Events	HSOPS- Persian 12/42 (complete) Emergency/ acute care, ICU, surgery, medicine, NR Nurses *n* = 2,995 Response rate: 51.1%	Retrospective nurse-estimate Incidence of AEs last year, 7-point scale 2,995 nurses 6 AEs: Pressure ulcer, patient falls, adverse drug events, surgical wound infection, complaints from patients or their family, infusion or transfusion reaction	Bivariate and multiple logistic regression models Hospital level 32 hospitals	^a^Increased scores of nine PSC dimensions were significantly associated with a reduced perception of AE rates in at least two out of six AEs ^a^*p* < *0.001*	**Fair**
Kline (2008) Canada [49] Patient Safety Culture/ Adverse Events	Patient Safety Culture 2005 Survey – Database 1/5 NR NR (reported as nursing leaders in primary study) *n* = 298 (408/417 in primary study) Response rate: 83%/ 72% (reported in primary study)	Register Regional Incident Reports in forms by any health staff 5,070 incident reports/ 3,093 non-incident reports Severity range 1–4 and contributing factors/ categorised as 7 incident types: Care and treatment, injury or death, falls, medication discrepancy, medication incident, test or results, vaccine	Hierarchical linear regression – multilevel Unit level 3 hospitals 40 units	^a^Resource intensity predicts incident severity level ^b^PSC predicts “adverse event severity” over “case resource intensity” ^*a*^*p* < *0.001* ^b^*p* < 0.05 *R*^*2*^ = *0.093*	**Fair**
Lee (2018) Canada [50] Organisational Safety Culture/ Adverse Events	HSOPS NS/7 NR Nurses *n* = 1,053 Response rate: NR	Retrospecitve nurse-estimate Incidence of AEs last year, 7-point scale 1,053 nurses 3 AEs: Medication error, patient falls with injury, urinary tract infection (+ quality of care)	Multilevel ordinal logistic and linear regression Pearson and Spearman correlation Hospital level 63 hospitals	^a^Increased Overall organisational safety culture was associated with reduced rates of reported medication errors, falls with injury and urinary tract infections ^a^Increased scores of Overall organisational safety culture increased the quality of care ^a^*p* < 0.05	**Fair**
Mardon (2010) US [51] Patient Safety Culture/ Adverse Events	HSOPS 12/42 (complete) NR NR *n* = 56,480 Response rate: 51%	Register HCUP at AHRQ – Patient safety indicators (PSI) NR 8 AEs: Complications of anesthesia, death in low mortality diagnostic related groups, failure to rescue, foreign body left in during procedure, transfusion reaction, birth trauma – injury to neonate, obstetric trauma vaginal delivery with or without instrument or with cesarean delivery. Composite score	Bivariate correlations and multivariate logistic regression Hospital level 179 hospitals	Increased score on following PSC dimensions were moderately associated with reduced PSI composite score: ^a^Frequency events reported ^b^Handoffs and transitions ^c^Management support for patient safety ^d^Organisational learning – continuous improvement ^e^Staffing ^f^Teamwork across units ^g^Teamwork within units ^h^Overall perceptions of patient safety ^i^ Supervisor/manager expectations and actions ^j^ Patient safety grade ^k^HSOPS composite average The PSC dimensions Communication openness, Feedback and communication about error and Number of events reported were not significantly correlated to PSI composite score Bivariate: ^1−3a,b,c,e,f,h,k^*p* < 0.001 ^d,g,i^*p* < 0.01 ^j^*p* < 0.05	**Fair**
McLinton (2019) Australia [52] Physical Safety Climate and Psychosocial Safety Climate/ Patient Incidents	Psychosocial safety climate – 12 4/12 (complete) NR Nurses, physicians, managers, others *n* = 436/ 60 teams (groups of individuals with an identifiable leader) Response rate: NR	Institutional incident safety system data NR Average number of incidents/ patient Any events causing harm or “near miss” accident (i.e. medication errors and falls)	Multilevel correlation and hierarchical linear model Pearson correlation Individual and group level 1 hospital	Increased ^a^Psychosocial safety climate composite scores and ^b^Burnout were significantly associated with reduced rates of patient incidents Increased Psychosocial safety climate composite scores were significantly associated with reduced scores of ^c^Burnout and increased scores of ^d^Engagement Increased Physical safety climate composite scores were significantly associated with increased scores of ^e^Burnout Increased scores in Psychosocial safety climate and Physical safety climate were significantly associated with reduced scores of ^f^Emotional demand, ^f^Bullying, ^f^Skill discretion ^a^*p* < .0.001 ^b^*p* < .0.01 ^c^ < 0.01- ind. level < 0.01- team level ^d^ < 0.001- ind. level < 0.05- team level ^e^ < 0.05- ind. level < 0.001- team level ^f^*p* < .0.01–0.001	**Fair**
Najjar (2015) Palestine [53] Patient Safety Culture/ Adverse Events	HSOPS – Arabic 12/42 (complete) Surgery/ medicine/ obstetrics Nurses, physicians, others *n* = 316 Response rate: 74%	Chart review Global Trigger Tool (GTT) 640 Records 54 Triggers	Bivariate regression Spearman *rho* correlation Unit level 2 hospitals 8 units	Increased scores in 8/15 PSC dimensions were significant associated with reduced AE rates: ^a^Aggregate safety culture ^b^Hospital management support ^c^Non-punitive response to error ^d^Open communication/ feedback received on error ^e^Teamwork within units ^f^Supervisor expectations and action promoting patient safety ^g^Organisational learning ^h^Patient safety grade ^a,d^ *p* < .0.001 ^b,e^*p* < 0.002 ^c^*p* < 0.020 ^f^*p* < 0.003 ^g^*p* < 0.011 ^h^*p* < 0.018	**Good**
Odell (2019) US [54] Hospital Safety Culture/ Surgical Outcomes	SAQ – modified + engagement surgeons 8/57 (complete) Surgery Nurses, physicians, others *n* = 871 Response rate: 47%	Register American College of Surgeons (ACS) NSQIP database NR 4 AEs: Risk-adjusted morbidity, mortality, DSM and unplanned readmission rates Morbidity measure captures cardiac arrest requiring resuscitation, myocardial infarction, ventilator dependence > 48 h, pneumonia, progressive renal insufficiency, acute renal failure, sepsis or septic shock, deep incisional, organ space, superficial surgical site infection, stroke/ CVA, unplanned intubation, urinary tract infection, dehiscence DSM includes complications in the morbidity measure except for ventilator dependence, superficial SSI, stroke/ CVA, and additionally includes venous thromboembolism	Linear regression and hierarchical logistic regression ^a^*p* < 0.007 ^b^* p* < 0.004 ^*c*^* p* < 0.23 ^d^* p* < 0.52 Hospital level 49 hospitals	Increased PSC composite scores were associated with reduced rates of ^a^postoperative morbidity and^b^ DSM No significant association between PSC and ^c^the risk of ^d^mortality or readmission	**Fair**
Olds (2017) US [55] Hospital Safety Climate/ Mortality	HSOPS + (named Multi-state Nursing Care and Patient safety study survey) NR/7 Emergency/ acute care Nurses *n *= 27,009 Response rate: 39% (non-responders assessed) (97)	Register Discharge records 852,974 patients 1 AE: In-hospital mortality	Bivariate correlation and multivariate logistic regression Hospital level 600 hospitals	^a^Increased PSC composite score was correlated with reduced mortality ^b^Perception of safety climate is not predictive of patient mortality beyond the Effect of nurse environments ^a^*p* < 0.001 ^b^*p* < 0.316	**Good**
Profit (2020) US [56] Safety Culture/ Quality of Care	SAQ 6/30 (complete) NICU Nurses, physicians, others *n* = 2,073 Response rate: 62.9%	Register CPQCC clinical data NR 9 AEs: Antenatal corticosteroids, hypothermia, pneumothorax, healthcare-associated infection, chronic lung disease, retinopathy screen, discharge on any human milk, growth velocity, mortality	Correlation tests Pearson *r* correlation ^*a*^*p* < 0.01 ^b^*p* < 0.05 Unit level 44 units	^a^Increased scores of Teamwork climate and ^b^Safety climate were correlated with a reduction in 1/9 of the metrics, healthcare associated infections (HAI)	**Good**
Quach (2021) US [31] Safety Climate/ Adverse Events	CESARS (ORCA`s organisational culture) 7/28 (+ 6/23) (complete) Outpatient/ homes Nurses, physicians, others *n* = 1,397 (first survey)/ *n* = 1,645 (second survey) Response rate: 26.4% and 27.7%	Register FY2017-FY2018 Minimum Data Set VHA 4 AEs: New/ worsened pressure ulcers, falls, major injuries from falls, catheter use	Bivariate logistic regression Group level 56 CLCs	Increased scores of Supervisor`s commitment to safety were associated with ^a^reduced rates of falls (clinicians) and ^b^reduced rates of catheter use (nurses) Increased scores of Environmental safety were associated with ^c^reduced rates of pressure ulcers (clinicians), ^d^reduced rates of major injuries from falls (nurses), and ^e^reduced rates of catheter use (nursing assistants) ^f^Increased scores of Global ratings were associated with higher level of catheter use for nurses and nursing assistants ^a,e^*p* < 0.05 ^b,c,d,f^*p* < 0.01	**Fair**
Rosen (2010) US [57] Hospital Safety Climate/ Safety Outcomes	PSCHO 11/42 NR Physicians, managers, others *n* = 9,309 Response rate: 50%	Chart review PSI software discharge records 13 AEs: Complications of anesthesia, decubitus ulcer, failure to rescue, iatrogenic pneumothorax, infection due to medical care, postoperative (po) fracture, po hemorrhage or hematoma, po physiologic and metabolic derangement, po respiratory failure, po pulmonary embolism or deep vein thrombosis, po sepsis, po wound dehiscence, accidental puncture or laceration	Linear regression Hospital and group level 30 hospitals	**No statistically significant association** between PSC overall scores and rates of PSIs or PSI composite rates Increased scores of individual dimensions were correlated with reduced rates of specific PSIs: Fear of blame and punishment for making mistakes with ^a^decubitus ulcer and ^b^postoperative pulmonary embolism or deep vein thrombosis Perception of lower psychological safety with ^c^failure to rescue Overall emphasis on safety with ^d^decubitus ulcer and ^e^iatrogenic pneumothorax ^acde^*p* < 0.05 ^b^*p* < 0.01	**Fair**
Shahian (2018) US [58] Hospital Safety Culture/ Mortality	HSOPS 12/42 (complete) Emergency/ acute care NR *n* = 257 hospital- surveys *n* = 834 average/ hospital Response rate: 54% (5–100%)	Register MEDPAR 1,609 patients 19,357 discharges 1 AE: Risk-adjusted mortality	Multivariate hierarchical logistic regression Hospital level 171 hospitals	**No statistically significant association** was found between PSC scores and rates of 30-day mortality Reported as OR	**Fair**
Singer (2009) US [59] Hospital Safety Climate/ Safety Performance	PSCHO 8/38 Emergency/ acute care Nurses, physicians, others *n* = 18,223 Response rate: 52%	Register MEDPAR- PSI 12 AEs: Complications of anesthesia, decubitus ulcer, iatrogenic pneumothorax, infection due to medical care, postoperative (po) hip fracture, po hemorrhage or hematoma, po physiologic and metabolic derangement, po respiratory failure, po pulmonary embolism or deep vein thrombosis, po sepsis, po wound dehiscence, accidental puncture or laceration	Multilevel logistic regression Hospital level 91 hospitals	Increased scores in the PSC dimension ^a^Fear of shame/ blame and ^b^Overall PSC were associated with reduced PSI composite rates ^ab^*p* < 0.05	**Fair**
Smits (2012) Netherlands [60] Patient Safety Culture/ Unintended Events	HSOPS – Dutch version named COMPaZ 11/40 Surgery/ medicine/ Emergency and acute care Nurses, physicians, managers, others *n* = 542 Response rate: 56%	Prospective reporting Staff wrote reports of all unintended events 1,885 Events 8 Classifications: Materials and equipment, diagnosis and treatment, medication, protocols and regulations, incorrect data and substitutions, collaboration with resident physicians and consultants, collaboration with other departments and other	Multilevel logistic regression Unit level 20 hospitals 28 units	Increased scores in 3/11 PSC dimensions, ^a^Nonpunitive response to error, ^b^Hospital management support and ^c^Willingness to report were significantly associated with reduced rates of unintended events (medication, materials/ equipment and collaboration with resident physicians/consultants) ^a,b^*p* < 0.01–0.05 ^c^*p* < 0.001–0.01	**Fair**
Steyrer (2013) Austria [61] Safety Climate/ Medical Error	VSCQ 4/40 ICU Nurses, physicians *n* = 734 Response rate: 41.4% (nurses) and 35.2% (physicians)	Prospective reporting Form to record predefined medical errors 48 h 378 patients 7 categories AEs: Administration of medication, unplanned dislodgement of airways, arterial lines, central venous catheters, urinary catheters, enteral nutrition probes, or drains. Error rate: rate of ratio affected by errors in an ICU/ total number of patients	OLS regression (Ordinary Least Squares) Unit level 57 units	Increased scores of following PSC dimensions were significantly associated with reduced AE composite rates ^a^Increased scores of Workloads increases the error composite rate, and ^b^ increased scores of Safety climate reduced the AE composite rate PSC scores were more associated with reduced AE composite rate than safety tools ^a^*p* < 0.01 ^b^*p* < 0.05	
Tawfik (2019) US/ California [62] Safety Climate and Strength/ Outcomes	SAQ 0/7 NICU Nurses, physicians, others *n* = 2,073 Response rate 62.9%	Register CPQCC clinical data 6,682 patients 4 AEs: LOS, infections, chronic lung disease and mortality	Logistic linear regression Unit level 44 units	Increased scores of Safety climate strength (the consistency of responses) were significantly associated with reduced ^a^LOS Safety strength and Safety climate predicted LOS more than Safety climate separately, and increased scores of Safety strength were associated with lower odds of infection, but not other secondary outcomes ^a^*p* < 0.001	**Good**
Thomas-Hawkins (2015) US [33] Patient Safety Culture/ Adverse Events	HSOPS – modified 2/5 Outpatient/ homes Nurses *n* = 422 Response rate 52%	Retrospective nurse-estimate Incidence of AEs last year – series of survey items, 7-point scale 422 Nurses 13 AEs: Medication error, complaints from patient/family, vascular access infection, vascular assess infiltration, hospital admission, skipped dialysis, shortened dialysis, dialysis hypertension, falls without injuries, falls with injury, bleeding from vascular access, emergency room use, vascular access thrombosis	Logistic regression Unit level From 47 states	Increased scores of Poor to failing patient safety grade were significantly associated with reduced rates of ^a^medication error, ^b^complaints from patient/ family, ^c^vascular access infection, ^d^hospital admission and ^e^skipped dialysis, ^f^falls without injuries, ^g^bleeding from vascular access and ^h^emergency room use Increased scores of Patient handoffs and transitions were significantly associated with reduced rates of ^i^vascular access thrombosis, ^j^complaints from patient/ family, ^k^skipped dialysis, ^l^ shortened dialysis, ^m^emergency room use, ^n^bleeding from vascular access, ^o^ vascular access infection, ^p^medication error and ^q^vascular access infiltration Increased scores of overall PSC were significantly associated with lower odds of frequent rates of medication errors by nurses, patient hospitalisation, vascular access infection, and patient complaints ^a,b,i,j,k,l^*p* < 0.001 ^c,d,e,m,n,o^*p* < 0.01 ^f,g,h,p,q^*p* < 0.05	**Poor**
Valentin (2013) Austria [63] Safety Climate/ Medical Error	VSCQ 5/53 (complete) ICU Nurses, physicians *n* = 2,563 Response rate: 41.5% (nurses) and 35.2% (physicians)	Prospective reporting Form to record predefined AEs 48 h 795 patients 2 AEs: Medication errors and dislodgement errors	Multivariate logistic regression Unit level 57 units	^a^Increased scores of Safety climate overall were significantly associated with reduced AE rates ^b^Increased scores of Workloads at patient level were statistically significantly associated with increased AE rates ^ab^*p* < 0.01	**Fair**
Wang (2014) China [64] Patient Safety Culture/ Adverse Events	HSOPS 12/ 42 (complete) Surgery, medicine, Emergency/ acute care, Intensive Care Unit Nurses *n* = 463 Response rate: 72.3%	Retrospective nurse-estimate Incidence of AEs last year, 7-point scale 463 nurses 7 AEs: Pressure ulcers, prolonged physical restraint, complaints from patient/family, medicine errors, infusion or transfusion reaction, patient falls, surgical wound infection	Bivariate and Multivariate logistic regression Unit and hospital level 7 hospitals 28 units	Increased scores of the following PSC dimensions were significantly associated with reduced rates of specified AEs: Organisational learning – continuous improvement with ^a^pressure ulcers, ^b^prolonged physical restraint and ^c^complaints from patient/family Frequency of event reporting with ^d^medicine errors and ^e^pressure ulcers Feedback and communication about error with ^f^pressure ulcer and ^g^infusion or transfusion reaction Hospital Management support for patient safety with ^h^medicine error and ^i^infusion or transfusion reaction Supervision expectations and actions promoting safety with ^j^complaints from patient/ family Non-punitive response to error with ^k^pressure ulcers Handoffs and Transitions with ^l^infusion or transfusion reaction ^a^*p* = 0.002 ^b^*p* = 0.019 ^c^*p* = 0.013 ^d^*p* = 0.021 ^e^*p* = 0.006 ^f^*p* = 0.037 ^g^*p* = 0.041 ^h^*p* = 0.006 ^i^*p* = 0.027 ^j^*p* = 0.029 ^k^*p* = 0.045 ^l^*p* = 0.034	**Fair**

Abbreviations: *PSC* Patient Safety Culture, *AE* Adverse Events, *SOS* Safety Organizing Scale, *US* United States, *NR* Not Reported, *AHRQ* Agency of Healthcare Research and Quality, *HSOPS* Hospital Survey of Patient Safety Culture, *SAQ* Safety Attitude Questionnaire, *OR* Operating Room, *TCI* Team Climate Inventory, *CVF* Competing Values Framework, *CALNOC* Collaborative Alliance for Nursing Outcomes, *HAPU* Hospital-Acquired Pressure Ulcers, *IRR* Incidence Rate Ratio, *NSQIP* National Surgical Quality Improvement Program, *SSI* Surgical Site Infection, *ASA*American Society of Anesthesiologists, *ICU* Intensive Care Unit, *OR* Odds Ratio, *PICCM* Project IMPACT Critical Care Medicine, *LOS* Length of Stay, *HCUP* Healthcare Cost and Utilization Project, *PSI* Patient Safety Indicators, *GTT* Global Trigger Tool, *ACS* American College of Surgeons, *DSM* Death or Serious Morbidity, *CVA* Cerebrovascular Accident, *NICU* Neonatal Intensive Care Units, *CPQCC* California Perinatal Quality Care Collaborative, *HAI* Healthcare Associated Infections, *CECARS* Community Living Center Employee Survey of Attitudes about Resident Safety, *ORCA* Organizational Readiness to Change Assessment, *VHA* Veterans Health Administration, *CLC* Community Living Centers, *PSCHO* Patient Safety Climate in Healthcare Organizations, *MEDPAR* Medicare Provider Analysis and Review File, *VSCQ* Vienna Safety Climate Questionnaire, *OLS* Ordinary Least Squares

The studies used a variety of concepts to illuminate PSC. ‘Safety culture’ was used in 56% and ‘Safety climate’ in 44% of the studies. Different concepts were also used to illuminate AEs, and the most used concepts were ‘Events’ in 32% of the studies and ‘Outcomes’ in 26%. Some of the concept`s definitions were interchangeable at both side of the association. More details on the variety of concepts and their prefixes used are presented in Table 3.

### Association between PSC scores and AE rates

A vast majority (*n *= 32) of the included studies were designed as cross-sectional cohort studies and only two studies measured the association in quasi-experimental intervention studies [35, 44]. Most of the included studies, 76%, demonstrated that increased PSC scores were associated with reduced AE rates [31, 33, 36–38, 41, 43–56, 59–64]. Around one-quarter of the included studies, 24%, found no association*,* i.e*.* reduced AE rates by increasing PSC scores [32, 34, 35, 39, 40, 42, 57, 58]*.* Studies indicating that increased PSC scores predicted reduced AE rates, were based on the association between overall PSC score *or* the scores of *some* of the PSC dimensions, and the composite AE rates *or* the rates of *some* of the AEs. Some studies suggested that the PSC scores of frontline personnel, nurses and physicians, were more strongly associated with reduced AE rates than the perceptions of managers and administrators [36, 57, 59].

To examine the statistical association between PSC scores and AE rates, 74% of the studies used a various number of measured PSC dimensions [31, 33, 34, 36–41, 43, 45, 46, 48, 49, 51, 53, 54, 56–62, 64], however two studies measured more dimensions than they included in the analysis [56, 61]. Many of these studies also used the ‘overall safety grade’ to measure the association [39, 43, 45, 48, 51, 53, 54, 57–60, 64], and some studies only utilised the ‘overall safety grade’ [35, 42, 52, 63], composite measure of selected items [44, 50, 55], single items and dimensions to analyse the association [36], or dominant dimension of PSC from one tool and the overall score from another tool to assess the association [32]. In 47% of the studies, the rates of predefined AEs were used to examine the statistical association with PSC scores [31–34, 37, 41–43, 45, 47, 48, 50, 54, 62–64], and 21% used the overall AE rates [36, 51, 52, 56, 57, 59, 61]. Adjusted estimated mortality or morbidity was used in 21% [35, 38, 40, 44, 46, 55, 58] and AE categories and scales in 9% of the studies [39, 49, 60].

The statistical analyses conducted to examine the associations were bivariate and/or multivariate, mostly regression analyses (logistics, linear or hierarchical) and correlation analyses, detailed in Table 3. The statistical association between PSC scores and AE rates was explored at different levels of the healthcare system. Analyses were conducted at unit level in 44% and at hospital level in 38% of the studies, others used group- or individual levels or combinations of levels as presented in Table 3. The measurement of PSC scores and AE rates was conducted within the same time frame in 48% of the included studies [31–35, 38, 42, 43, 47–51, 61, 63, 64]. Fifteen per cent of the studies measured AE rates after the measurement of PSC scores [37, 45, 52, 59, 60], and 6% of the studies measured AE rates after and during the PSC measurement [40, 41]. Other studies, 9%, measured AE rates before PSC measurement [39, 53, 55], and 15% of the studies measured AE rates before, during and after the PSC score measurement period [46, 54, 56, 58, 62]. Some of the studies did not report the time frame for both PSC and AE measurements [36, 44, 57]. The length of the study period represented additional variation in the included studies. The study periods ranged from PSC measurements combined with prospective observed AE rates over 48 h [61, 63] or two weeks at each unit [42], PSC measurements combined with retrospective nurse-estimates over 3–4 months [33, 34, 43, 47, 48, 50, 64], to AE rates from chart reviews collected over four years and AE rates from registers collected over three years [38].

### Research methodology

All studies used a cross-sectional survey to measure PSC. The majority of the studies used the tools HSOPS (38%) and SAQ (29%) to measure PSC, and most of these studies used the complete tool, however some used translated og modified versions. Other studies measured selected items from HSOPS and/ or SAQ. Nurses’ and physicians’ perceptions of the PSC were, respectively, assessed in 79% and 59% of the included studies. *Others* were assessed in 44% of the studies and managers in 15% of the studies. *Others* included other clinical or administrative professionals in the healthcare. One study reported nurses’ PSC scores to be lower than those of surgeons [36], and another study suggested that hospital administrators have more positive perceptions of PSC than frontline personnel such as nurses and physicians [54]. Details of the PSC measurement are presented in Table 3.

The selected scientific papers demonstrated a variety of approaches to examining AE rates. Most of the studies employed a retrospective approach to measure AE rates (82%), and only a few, 18%, employed a prospective approach. Most of the included studies, 35%, used registers to obtain AE rates, 26% did chart reviews, 21% were based on nurse-reported estimates and 18% utilised the prospective staff`s reporting. Registers for obtaining AE rates were nationally based [31, 37, 38, 46, 51, 54], regional/state [49, 56, 62] or from institutional registers [52, 58, 59]. The details of the AE measurement are presented in Table 3.

### Strengths and limitations of the included studies

The key questions in the critical appraisal of the included studies were measurement of PSC scores and AE rates, response rate and adjustment for confounding variables. The critical appraisal resulted in 71% of the included studies being rated as ‘Fair’, 21% being rated as ‘Good’, and 9% being rated as ‘Poor’. The rating of the studies is visually presented in Table 3.

Sixty-five per cent of the studies reported complete and validated use of the PSC tool [31, 34, 37–43, 46, 48, 51, 53, 54, 56–61, 63, 64]. Other studies reported on reliability by measuring the internal consistency using Cronbach`s alpha [32, 33, 35, 45, 47, 49, 50, 52, 55]. The study using focus groups to select items from HSOPS and SAQ was the only study that did not report the validity or the internal consistency of the tool used [36].

For AE measurement, almost half of the utilised registers in the included studies were reported as reliable and validated systems [51, 54, 56, 58, 59, 62]. The AE measurement procedures from other registers were well described and clearly defined, but not reported as validated for the intended use [31, 38, 46, 49]. Another register had well-described definitions and was based on voluntary reporting [37]. One study that obtained AE rates from their mandatory safety reporting system did not define the AEs in detail [52].

Half of the studies obtaining AE rates from chart reviews used standardised procedures, AE definitions and trained staff to identify AEs in records [36, 39, 40, 45, 55]. However, two studies used a standardised procedure but did not report who conducted the chart reviews [35, 44]. One of these combined chart reviews and constructed internal validation through dialogue with clinicians [44]. Additionally, internal validation by consulting clinicians was used in one observational study using a chart review as a supplementary methodology, however, the method was not externally validated [42]. Another study documented the inter-rater reliability of their procedure [40]. Automated tools such as ‘Agency for Healthcare Research and Quality Patient Safety Indicator software’ and the GTT were reported as validated [53, 57]. The systematic prospective tools and the retrospective nurse-estimates for measuring AE rates were not considered valid by the studies themselves, although the data collection was described as standardised.

The response rate of PSC surveys ranged from 26.4%—97.7%. A few of the studies had a response rate < 50% (18%). Some of the included studies did not report the response rate, either the sample size. The sample size ranged from 184 participants to 214,338 participants, and 21% of the studies had a sample size < 500. The distribution of the sample in 15% of the studies resulted in an average sample size of < 10 participants per unit/hospital (*n* = 5), and 35% had a distribution of the sample resulting in an average sample size of < 20 participants per unit/hospital.

Most of the studies 82%, described how they measured and adjusted for key potential confounding variables. Some studies did not measure and adjust for confounding variables, or we could not determine whether this was done [33, 35, 37, 44, 53, 63].

## Discussion

We aimed to summarise the evidence on the association between PSC scores and AE rates in healthcare services, to map the characteristics and applied research methodology, and to study the strengths and limitations of the included studies. Recent evidence on the statistical association between PSC scores and AE rates reflects the increasing priority assigned to this topic over the last twenty years in response to the Institute of Medicine`s call for global and comprehensive efforts to improve the quality of healthcare [6, 10]. However, the included studies mainly originate from the US, Canada and Europe, and the evidence on the association is primarily conducted in-hospital. Hence there is a lack of studies from low- and middle-income countries, and a lack of studies conducted in primary care.

Most of the included studies demonstrated that increased PSC scores were statistically significantly associated with reduced AE rates. This is in line with previous reviews reporting mostly significant associations between PSC scores and patient safety outcomes [21, 65, 66]. The review assessing the association between ‘Organisational and workplace culture’ and patient outcomes, found that 74.2% of the evidence reported positive or mixed positive associations between culture and patient outcomes [21]. This finding is closely related to the evidence in this review and underlines the importance of promoting a culture for patient safety. As demonstrated in the Table 3, ‘Teamwork climate’ and ‘Safety climate’ tended to be the most frequent dimensions associated with reduced AE rates. This finding supports a review demonstrating that teamwork and communication training improves PSC scores and suggests that improving these dimensions may reduce AE rates [67]. Additionally, these finding rise the question about the concepts of ‘Patient safety culture’ and ‘Patient safety climate’. Previous reviews suggest that PSC questionnaires capture ‘Patient safety climate’ and the tangible themes, and not the PSC and intangible themes under the surface [17, 68]. Increased scores in the dimensions ‘Leadership’s perception and Action promoting patient safety’, ‘Management support’, ‘Communications openness and learning’, and ‘Non-punitive response to errors’, were also associated with reduced AE rates in the included studies. This resonates with a comprehensive review emphasising senior leadership as key to accountability for safety culture [69].

Fewer studies found that increased PSC scores did not reduce AE rates, and this is supported by previous research on the association between PSC and quality outcomes [19, 70]. However, one of the included studies that demonstrates no or an inverted result on AEs, reported that increased PSC scores were associated with a reduction in ‘Intercepted near-misses’ [39], which previous research indicates may be related to an actual reduction in “Near misses” [71]. These findings support increased PSC as a proxy for improved patient outcomes and better quality of care. Another of the studies demonstrating mixed results reported increased scores of specific PSC dimensions being associated with reduced rates of specific AEs [40, 57]. A previous review of the PSC literature identifies semantic inconsistencies, infrequent use of theory, limited discussion of the use of instruments, methodological variation in research on the relationship between safety culture and patient safety and quality of care outcomes [70]. Previous reviews and the discrepancy in the evidence on the association between PSC scores and AE rates in this review, make it reasonable to study the utilised methodology to further understand the discrepancy and improve research on the topic.

Despite the fact that all the included studies measuring PSC utilised a cross-sectional approach, there is great variation in conceptualisation, and whether the use of the tools was statistically validated for their specific use. There were some discrepancies between how authors utilised the PSC tools beyond the original complete tool and the composite scores described in the tools` guidelines. Only 41% the studies used the complete, validated and recommended tools HSOPS and SAQ [16, 72] to capture more dimensions of the PSC measurement. We suggest that these variations may affect the measurement of association with the AE rates in both directions, and thus reduce the validity of the measured association. Additionally, according to the results of included studies assessing the PSC measurement at group level, variations due to included groups of healthcare professionals and specialties can also affect the results. AEs related to surgery make up one of the three most common types of in-hospital AEs [4, 73], which may thus influence the association with PSC. Despite this, some of the included studies conducted in surgical contexts also reported finding no association [34, 35, 40]. Efforts to assess and understand PSC scores’ association with AE rates in a surgical context are relevant for further research. Whether the PSC measurements are utilised at hospital, unit or group level will likely affect the association with AE rates. It is known that PSC varies most between units within the same hospital due to organisational processes and structures [72, 74, 75]. Unit characteristics and work environment factors such as improvements, patient-centred care and quality are predictors of safety climate [76].

Representativeness is important for reliable interpretations. Studies with small samples sizes distributed in a high number of units or hospitals may not be powered to detect an effect of PSC on AEs. In addition, Pronovost and Sexton [72] recommend a response rate above 60% to capture the culture in an organisation rather than opinions. In this review, 62% of the studies report a response rate below 60%, thus this may affect the reliability of the PSC measurement and the association with AE rates. From the studies not demonstrating an inverse association between PSC scores and AE rates, 62.5% reported a response rate below 60% or did not report the response rate. The percentage for studies demonstrating an inverse association between PSC scores and AE rates was 61.5%. The evidence on PSC measurements in this review adds weight to the call for a more uniform, complete and validated approach to measuring and reporting PSC [17], measurements at unit or group level [72, 74], and a response rate above 60% as recommended [72].

Measurements of patient AE rates in the included studies display a great diversity of methodology, including conceptualisations, tools, validation criteria used, types of AEs assessed, and how the rates are used to measure association with the PSC scores. A previous review demonstrates that measured AE rates in the operating room depend on the method used. This review find that direct observations detect higher AEs rates than surveys, incident reports and reviews of patient charts [2]. De Vries et. al [3] demonstrates that a limitation of retrospective chart reviews was that the quality of data collection depends on documentation and interobserver variability [3]. Moreover, GTT and HMPS as manual methods for chart reviews are reported as reliable, however the inter-rater agreement increased when studies used a small group of reviewers [77]. The manual method of using the GTT tool is further developed and an automatic method for detecting triggers that indicate AEs is validated [78]. Reporting bias due to the prospective self-reporting may affect the results and may be affected by the Hawthorne effect [32, 41, 42, 60, 61, 63]. Hence, reporting bias and the Hawthorne effect may influence the retrospective nurse-estimated AE rates. The culture and supportive leadership for reporting and continuously learning from AEs may increase AE rates [79], and thus, if not adjusted for, this method of measuring AE rates may bias the results. Moreover, in 38% of the studies, AE rates were found by utilising administrative registers. Registers might be a useful method due to the opportunity for larger samples and the possibility to extract data at unit level [80]. However, less than half of the registers used in the included studies reported on their validity for the intended use. The available evidence on comprehensive, prospective national-level data on in-hospital AE rates is limited, and there is a lack of reporting on the validation of using the registers for AE data, thus, more transparent reporting is called for regarding studies using registers for AE data collection [4]. More standardised procedures are needed for validated identification, measured and reported AE rates [2, 4, 9].

We found studies that included other process variables in their assessment of the association between PSC and AE. Higher scores of ‘Patient safety competencies’ and ‘Safety training’ were reported to contribute to lower AE rates [42, 43]. Another included study found that reduced AE rates were associated with increased ‘Nurse work environment’ scores, described as enough staff to get the work done and provide quality of care, and the opportunities to discuss patient care and support colleagues [55]. A recent review reports a significant correlation between ‘Staff engagement’ and both PSC scores and AE rates, increased engagement as a cost-effective means of enhancing patient safety, and the health services’ need for a competent and engaged workforce of sufficient number [22]. Sexton et al. [81] have developed SAQ into ‘Safety, Communication, Operational Reliability, and Engagement’ (SCORE), where the main additions are ‘Staff burnout’ and ‘Resilience’. These dimensions are related to patient outcomes and are found to be critical to sustainable quality improvement [81]. Thus, SCORE captures more information related to processual factors and may contribute to providing more insights into the association between PSC and AEs.

We found indications that structural variables may have contributed to the finding that 24% of the studies did not yield reduced AE rates in association with higher PSC scores. One study found that the structural variable ‘Resource intensity’, i.e. resource allocation and patient case weighting, was related to the severity of AEs [49]. Increased ‘Hospital-level nurse-to-patient-ratio’ scores were reported to be significantly correlated with shortened length-of-stay, reduced readmission within 7 days and mortality [82]. Further, single studies reported that increased scores on the HSOPS dimension ‘Staffing’ were statistically significantly correlated with reduced AE rates [37, 48, 51, 60], increased ‘Workload’ was statistically significantly correlated with increased error composite rate and mortality [38, 42, 61], and increased ‘Rationing of nursing level scores’, described as nurses lack of possibility to act as needed, was statistically significantly correlated with reduced rates of specific AEs [34]. The latter study reported that ‘Nurse-to-patient-ratios’ were not significantly correlated with AE rates, and called on hospital units to monitor and balance the ‘Rationing of nursing level’ and education levels according to patients` characteristics [34]. Another included study reported a robust association between PSC scores and the ‘Structure of care delivery’ as ‘Skill mix’, ‘Contract workers’, ‘Patient ratio per licensed staff member’, ‘Turnover’ and ‘Workload intensity’, and between PSC scores and ‘Fall protocol’ as a measure of process [37].

Precautions must be taken regarding appropriate time frames for data collection on PSC and AEs due to other processual and structural variables. PSC measurement before AE measurement may influence the results because of the Hawthorne effect. Organisational processes may change PSC over time, however structural changes in an organisation and disruption due to leadership changes may negatively impact PSC more quickly [83]. Additionally, a time frame over years may be influenced by changes in other processual and structural factors affecting psychological and physical work environment in an organisation, and thus may, be liable to bias the results [76]. Determining the appropriate time frame can be difficult. Hence, precautions must be taken when designing studies on the association between PSC scores and AE rates.There is a need for a broader insight into the context, processual and structural variables, and how these can mediate and strengthen the inverse relationship between PSC scores and AE rates.

### Strengths and limitations of this review

Our study has several limitations. Firstly, there is a risk of both selection and publication bias due to the large variety of methodological approaches and operationalisation of the concepts that our search strategy might not fully scope. This also includes that we have not searched grey literature [25]. Secondly, as we searched for the measurements of PSC and AE and the association between these variables, we excluded studies with a qualitative approach. Thirdly, the study protocol was not prospectively published, which implies reduced transparency. Fourth, we searched for single peer-reviewed articles and excluded conference abstracts and dissertations. This may result in the exclusion of relevant evidence. However, studies of dissertations within the inclusion criteria should be captured by the search strategy.

This study has several strengths. Firstly, a comprehensive search strategy was conducted and guided by a senior librarian with expertise in medical literature searches and reviews. The search used broad concepts, proximity operators and several relevant databases. The selected databases were multidisciplinary to identify such studies. Secondly, a blinded screening process ensured eligibility, and a detailed charting form ensured the included studies were mapped and summarised. Thirdly, the PRISMA-ScR checklist was utilised throughout the research process, and, as a result of the findings, we conducted a critical appraisal to answer the aim of this scoping review.

## Conclusions

Most of the studies demonstrated that an increased PSC score is statistically associated with reduced AE rates. However, a quarter of the studies contradicted the main findings. The main characteristics of the evidence are that most of the studies are conducted as multicentre studies, in-hospital, in high-income countries, and are measuring the perceptions of nurses and physicians. The evidence on the association indicates a need for more uniform PSC and AE measurements utilising well-defined concepts, complete and validated tools, transparent reporting and data collection within appropriate time frames and study level to provide studies eligible for meta-analysis and synthesis, hence a better understanding of the relationship between PSC and AEs in healthcare. Longitudinal prospective research at group and unit level, especially in the surgical context, combined with qualitative approaches for a broader understanding of the context and the central concepts, may be valuable. Consequently, high-quality quantitative research can provide increased insights into the association and confounding variables and may identify interventions to reduce AEs and inform quality healthcare improvement projects.

## Supplementary Information

**Additional file 1. **Study protocol

**Additional file 2. **Charting form

**Additional file 3. **Critical appraisal

**Additional file 4. **PRISMA-ScR checklist

## Data Availability

The data that constitute this review are included in the published article.

## Abbreviations

AE: Adverse events
GTT: Global trigger tool
HMPS: Harvard medical practice study
HSOPS: Hospital survey of patient safety culture
PSC: Patient safety culture
SAQ: Safety attitude questionnaire
