# Supplementary material for: The association between patient safety culture and adverse events – a scoping review
Source: BMC Health Serv Res. 2023 Mar 29;23:300. doi: 10.1186/s12913-023-09332-8 (PMC10053753; doi:10.1186/s12913-023-09332-8)
Supplement: Supplementary file 1 — Additional file 1. Study protocol [file 12913_2023_9332_MOESM1_ESM.docx]

**Title: The association between Patient safety culture and Adverse Patient Events**

**Background**

The concept of safety culture became central in various businesses after the Chornobyl disaster in 1986. Since then, the interest in patient safety culture in healthcare systems has increased (1). Guldenmund (2) presents research on safety culture and safety climate. Lee`s explicit definition, dated 1966, describes that: “*The safety culture of an organization is the product of individual and group values, attitudes, perceptions, competencies, and patterns of behaviour that determine the commitment to, and the style and proficiency of, and organisation`s health and safety management*” (2). Measurements of patient safety culture may identify levels of the staff`s perceptions of patient safety culture and variations across the organizational units. Valid measurement can predict variability in risk related to tasks, work environment, staff behavior, and patient outcome. It is often measured using a survey or a questionnaire (3). Colla, Bracken and Kinney (4) compared characteristics, dimensions, psychometrics and use of patient safety climate surveys. They found nine patient safety climate surveys. The surveys and the strength of psychometric testing vary considerably, but nearly all surveys covered the five dimensions of leadership, policies and procedures, staffing, communication, and reporting (4). The most used instruments for measuring patient safety culture are The Safety Attitudes Questionnaire (SAQ) and the Agency for Healthcare Research and Quality (AHRQ) Hospital Survey on Patient Safety Culture (HSOPS) (5).

Medical errors resulting in adverse events (AE) were highlighted when The Institute of Medicine (IOM) published the report “*To err is human*” (6). The report estimates the national costs of preventable adverse events to be between $17 and $29 billion, and medication errors alone account for over 7000 deaths. The report’s goal is to stop the cycle of inaction and make a comprehensive approach to improve patient safety. Kohn et al. (6) define safety as “*freedom from accidental injury*”, and present that the healthcare system has to prevent errors at all levels. The report clarifies that the healthcare system needs to learn from the analysis of errors (6). AEs and lack of patient safety and quality in the healthcare system are global concerns. World Health Organization (WHO) reported in 2015 that hospitalizations in low- and middle-income countries (LMICs) lead to 134 million AE each year, and deaths related to unsafe care in LMICs constitute 10 and 15 per cent of the total deaths. Poor-quality care is not limited to LMIC. Organization for Economic Co-operation and Development (OECD) found that 15 per cent of all hospital costs in OECD countries are associated with AE (7). “Harvard method” and “Global Trigger Tool (GTT)” are methods designed to provide data on the frequency and types of AEs (8).

Donabedian (9) developed a framework for evaluating quality in medical care. Quality is a “*reflection of values and goals current in the medical care system and in the larger society of which it is a part*” and represents a complex phenomenon in health care (9). According to Donabedian, the quality and assessment of medical care are multidimensional, including the three dimensions, process, structure and outcome. There is complexity and ambiguity in the relationship between the process and the structure, and the assessments are even more complicated when the dimensions of process and structure are related to the outcomes. (9). This will constitute the theoretical framework in this scoping review. Colla et al. (4) point out the need for more research to understand the relationship between the measures of patient safety culture and patient outcomes. The available evidence on comprehensive, prospective national-level data on in-hospital adverse events is limited (10). DiCurrio (11) indicates some correlations between patient safety culture and outcomes. The review includes studies measuring patient and family satisfaction and nurse-sensitive patient outcomes. Studies determining healthcare personnel’s perceptions are not included. DiCurrio recommends further research using a cross-sectional design and meta-analysis of the available studies to strengthen the connection between specific patient outcomes and patient safety culture. (11). Groves (12) devised a meta-analysis on these connections in acute-care hospitals. The available empirical research was limited, but five small pilot meta-analyses were conducted. There was no significant relationship between safety culture and the outcomes of pressure ulcers, medication errors, nurse-sensitive outcomes and postoperative outcomes (12).

Janes et al. (13) identified a small but consistent and significant relationship between staff engagement, patient safety culture, and adverse events. Increasing staff engagement can offer an impactful and cost-effective means of enhancing patient safety outcomes. Further research is needed to determine individual, team and unit relationships. The literature on safety culture and errors should have a multidisciplinary focus. In light of the recent focus on patient safety culture, it will be significant to evolve this science into safety outcomes (13). Lee et al. (14) conducted a literature review to examine the relationship between safety culture and patient safety and quality of care outcomes. The review identified a nonsignificant and inconsistent relationship. Literature on the association consists of semantic inconsistencies, infrequent use of theory, limited discussion of the use of the instruments, and methodological variations (14).

This scoping review aims to map and summarize the evidence on the associations between patient safety culture and adverse patient outcomes in health care services, map the research methods and identify knowledge gaps.

**Methods**

**Scoping review**

We plan to conduct a scoping review based on the framework developed by Arksey and O`Mallye (15). We will utilize the PRISMA-ScR (PRISMA extension for Scoping Reviews) checklist and explanation for complete and transparent conducting and reporting of the scoping review (16). A scoping review is an adequate methodological approach in this study because the research questions are broad compared to a question answered in a traditional systematic review. The rationale is to map the evidence and examine how research is conducted on a certain topic, indicating scoping review as the appropriate approach to answer the research question (17).

**Search strategy**

An introducing literature search of the Medline (Ovid) and Embase databases was conducted in January 2021 using *“patient safety culture” AND “medical error*” OR “adverse event*”. “Medical error*”* is a MESH term, but the other search terms were used as keywords. The search included studies in English and Scandinavian languages without limitation on year. The first search indicated that the search strategy indicated relevant literature on the topic. To ensure the quality of the main search, we obtained guidance from an experienced research librarian. The search strategy ended broadly to identify all relevant, peer-reviewed studies. The search in Medline (Ovid) is constructed *((patient safety) adj10 culture).tw,kw,kf AND medical errors OR adverse event* OR adverse fail* OR adverse outcome* OR clinical complications* limitation language*. We will conduct the same search in the databases Embase, PsykInfo, Cinahl, Cochrane Library and Epistemonikos. We will search in the interprofessional databases Web of Science and Business Source Elite to identify relevant studies conducted from organizational and management perspectives. We plan to perform a search in Google scholar to identify some grey literature.

**Screening**

After duplicate control in Endnote and Rayann, MV and SOD will conduct a blinded, independent screening of the results to select sources in Rayyan Software. The first screening will be based on titles and abstracts. We will search for single studies exploring the association between patient safety culture and patient adverse outcomes empirically. The studies included must perform a measurement of patient safety culture dimensions. We will compare the screening test after the first hundred records to ensure a common understanding of which studies illuminate the review`s scope. When there is a conflict about whether the record should be included, we will discuss it until consensus is achieved. After screening based on the title and abstract, we will include records for further reading the articles in full text. As the first screening, this screening will be conducted independently in Rayyan. The screening- and eligibility- process will be presented in a flow chart.

**Data Charting**

We will use a well-constructed Excel data charting form to answer the review's scopes. From the selected studies, we will chart the first author, origin, single- or multicenter stadium, the purpose of the stadium, intervention, tools to measure patient safety culture and outcomes, what outcomes were measured, analysis methods, analysis related to the association, key findings, knowledge gaps identified, and relevant articles in the reference list.

The results will be summarized and presented descriptively and in tables.

**Reference List**

1. Flin R, O'Connor P, Crichton M. Safety at the sharp nd : a guide to non-technical skills. Aldershot: Ashgate; 2008.

2. Guldenmund FW. The nature of safety cultre : a review of theory and research: Safety Culture & Climate. Safety science. 2000;34(1-3):215-57.

3. Weaver SJ, Lubomksi LH, Wilson RF, Pfoh ER, Martinez KA, Dy SM. Promoting a culture of safety as a patient safety strategy: a systematic review. Annals of Internal Medicine. 2013;158(5 Pt 2):369-74.

4. Colla J, Bracken A, Kinney L, Weeks W. Measuring patient safety climate: a review of surveys. BMJ Quality & Safety 2005;2005;14:364-366.

5. Halligan M, Zecevic A. Safety culture in healthcare: a review of concepts, dimensions, measures and progress. BMJ Quality & Safety 2011;2011;20:338-343.

6. Kohn LT, Corrigan J, Donaldson MS. To err is human : building a safer health system. Washington, D.C: National Academy Press; 2000.

7. National Academies of Sciences EaM. Crossing the Global Quality Chasm: Improving Health Care Worldwide. Washington, DC: The National Academies Press; 2018.

8. Unbeck M, Schildmeijer K, Henriksson P, Jürgensen U, Muren O, Nilsson L, et al. Is detection of adverse events affected by record review methodology? an evaluation of the “Harvard Medical Practice Study” method and the “Global Trigger Tool”. Patient Safety in Surgery. 2013;7(1):10.

9. Avedis D. Evaluating the Quality of Medical Care. Milbank Mem Fund Q. 1966;44(3):166-206.

10. Makary MA, Daniel M. Medical error—the third leading cause of death in the US. BMJ. 2016;353:i2139.

11. DiCuccio MH. The Relationship Between Patient Safety Culture and Patient Outcomes: A Systematic Review. Journal of patient safety. 2015;11(3):135-42.

12. Groves PS. The Relationship Between Safety Culture and Patient Outcomes: Results From Pilot Meta-Analyses. West J Nurs Res. 2014;36(1):66-83.

13. Janes G, Mills T, Budworth L, Johnson J, Lawton R. The Association Between Health Care Staff Engagement and Patient Safety Outcomes: A Systematic Review and Meta-Analysis. Journal of Patient Safety. 2021;Publish Ahead of Print.

14. Lee SE, Scott LD, Dahinten VS, Vincent C, Lopez KD, Park CG. Safety Culture, Patient Safety, and Quality of Care Outcomes: A Literature Review. Western Journal of Nursing Research. 2019;41(2):279-304.

15. Arksey H, O'Malley L. Scoping studies: towards a methodological framework. International journal of social research methodology. 2005;8(1):19-32.

16. Tricco AC, Lillie E, Zarin W, O'Brien KK, Colquhoun H, Levac D, et al. PRISMA Extension for Scoping Reviews (PRISMA-ScR): Checklist and Explanation. Annals of Internal Medicine. 2018;169(7):467-73.

17. Munn Z, Peters MDJ, Stern C, Tufanaru C, McArthur A, Aromataris E. Systematic review or scoping review? Guidance for authors when choosing between a systematic or scoping review approach. BMC Med Res Methodol. 2018;18(1):143.
